# Selected Kefir Water from Malaysia Attenuates Hydrogen Peroxide-Induced Oxidative Stress by Upregulating Endogenous Antioxidant Levels in SH-SY5Y Neuroblastoma Cells

**DOI:** 10.3390/antiox10060940

**Published:** 2021-06-10

**Authors:** Muganti Rajah Kumar, Swee Keong Yeap, Han Chung Lee, Nurul Elyani Mohamad, Muhammad Nazirul Mubin Aziz, Melati Khalid, Mas Jaffri Masarudin, Adam Thean Chor Leow, Janna Ong Abdullah, Noorjahan Banu Alitheen

**Affiliations:** 1 Department of Cell and Molecular Biology, Faculty of Biotechnology and Biomolecular Sciences, Universiti Putra Malaysia, Serdang 43400 UPM, Selangor Darul Ehsan, Malaysia; drmuganti@gmail.com (M.R.K.); elyani.mohamad@ums.edu.my (N.E.M.); muhammadnazirulmubin@gmail.com (M.N.M.A.); masjaffri@upm.edu.my (M.J.M.); adamleow@upm.edu.my (A.T.C.L.); janna@upm.edu.my (J.O.A.); 2 China-ASEAN College of Marine Sciences, Xiamen University Malaysia, Sepang 43900, Selangor Darul Ehsan, Malaysia; skyeap@xmu.edu.my (S.K.Y.); l.han.chung@gmail.com (H.C.L.); 3 Biotechnology Research Institute, Universiti Malaysia Sabah, Kota Kinabalu 88400, Sabah, Malaysia; 4 Department of Biomedical Sciences, Faculty of Medicine and Health Sciences, Universiti Putra Malaysia, Serdang 43400 UPM, Selangor Darul Ehsan, Malaysia; melati@upm.edu.my; 5 UPM-MAKNA Cancer Research Laboratory, Institute of Bioscience, Universiti Putra Malaysia, Serdang 43400 UPM, Selangor Darul Ehsan, Malaysia

**Keywords:** kefir water, neurodegeneration, oxidative stress, antioxidant, neuroprotection

## Abstract

Kefir, a fermented probiotic drink was tested for its potential anti-oxidative, anti-apoptotic, and neuroprotective effects to attenuate cellular oxidative stress on human SH-SY5Y neuroblastoma cells. Here, the antioxidant potentials of the six different kefir water samples were analysed by total phenolic content (TPC), total flavonoid content (TFC), ferric reducing antioxidant power (FRAP), and 2,2′-diphenyl-1-picrylhydrazyl radical (DPPH) assays, whereas the anti-apoptotic activity on hydrogen peroxide (H_2_O_2_) induced SH-SY5Y cells was examined using MTT, AO/PI double staining, and PI/Annexin V-FITC assays. The surface and internal morphological features of SH-SY5Y cells were studied using scanning and transmission electron microscopy. The results indicate that Kefir B showed the higher TPC (1.96 ± 0.54 µg GAE/µL), TFC (1.09 ± 0.02 µg CAT eq/µL), FRAP (19.68 ± 0.11 mM FRAP eq/50 µL), and DPPH (0.45 ± 0.06 mg/mL) activities compared to the other kefir samples. The MTT and PI/Annexin V-FITC assays showed that Kefir B pre-treatment at 10 mg/mL for 48 h resulted in greater cytoprotection (97.04%), and a significantly lower percentage of necrotic cells (7.79%), respectively. The Kefir B pre-treatment also resulted in greater protection to cytoplasmic and cytoskeleton inclusion, along with the conservation of the surface morphological features and the overall integrity of SH-SY5Y cells. Our findings indicate that the anti-oxidative, anti-apoptosis, and neuroprotective effects of kefir were mediated via the upregulation of SOD and catalase, as well as the modulation of apoptotic genes (Tp73, Bax, and Bcl-2).

## 1. Introduction

Neurodegenerative diseases (NDD) affect millions of people and cause 17.1% of total disability-adjusted life-years for all causes and ages worldwide [1,2]. They are mainly due to the persistence of chronic oxidative stress and neuroinflammation. These are also the key factors in the maintenance and development of the neurodegenerative process in these disease conditions including, but not limited to, Alzheimer’s disease, Parkinson’s disease, and amyotrophic lateral sclerosis. The state of the oxidative stress can be characterised by an increase in the level of reactive species such as hydrogen peroxide (H_2_O_2_), and a decrease in, or incompetence of, the antioxidant molecules to eradicate free radicals [3,4]. Hydrogen peroxide can damage DNA and oxidise lipids and proteins under chronic oxidative stress. Although a low concentration of H_2_O_2_ is a potent mitogen in a number of cell types, beyond the threshold level, its oxidisable effects often result in cell damage and apoptosis, causing human disorders [4,5,6,7]. This reaction usually takes place in the brain neuron cells as they are highly sensitive, require high energy, and are niduses for many peroxidisable molecules [8,9]. Hence, the accumulation of H_2_O_2_ in the neuron cells spontaneously mediates apoptosis, damaging the neurons and causing severe loss in morphology and function resulting in a progressive degeneration in cognition and memory impairment [4,10]. Interestingly, several studies revealed that protective factors, such as antioxidants, can prevent H_2_O_2_-induced oxidative damages [4,5,11,12]. 

Kefir is a self-carbonated, refreshing fermented drink that has been consumed over hundreds of years for its various health benefits including its antioxidant, anti-inflammatory, and neuroprotective effects [13,14]. It is originated from communities in the Balkans in Eastern Europe and the Caucasian mountains [14]. Kefir has been consumed extensively as a probiotic drink, especially in Europe, Russia, and Southwest Asia as it is believed to promote good health. The beverage has a unique flavour due to its viscous texture with an acidic and tart taste and low levels of alcohol and other fermentation flavour products [13,14]. In addition, it also has extensive uses in food products worldwide, including sourdough bread, beer, white pickled cheese, and whey-based kefir beverages.

The feature that distinguishes kefir from other cultured products is the presence of various kinds of microbial flora, including the probiotic potential Lactobacillus species such as *Lactobacillus acidophilus*, *Lactobacillus casei*, and *Lactococcus lactis* subsp *Lactis* [14,15,16,17]. In kefir grains, several species of bacteria and yeasts are surrounded and immobilised by kefiran, a polysaccharide matrix (a water-soluble branched glucogalactan) where they coexist in symbiotic association [13,14]. The microbial species that coexist predominantly in kefir grains are lactic acid bacteria, acetic acid bacteria, bifidobacteria, and yeasts [15,16]. Kefir grains found in different parts of the world vary in their microbial composition, contributing to their different bioactive potencies. This is mainly due to the different culture maintenance methods and substrates used in the fermentation process. The major metabolites produced during kefir fermentation are lactic acid, acetic acid, ethanol, carbon dioxide, and smaller amounts of acetate, mannitol, and glycerol [18,19]. Esters such as isoamyl acetate, ethyl octanoate, ethyl hexanoate, and ethyl decanoate are also produced as an aromatic product of kefir fermentation. Moreover, this microbial species has various capabilities of synthesising amino acids including tryptophan, methionine, tyrosine, and histidine, as well as vitamins, pyridoxal phosphate, and cofactor biosynthesis by converting other amino acids found in the figs and other fruits added during fermentation [18]. These metabolites in the kefir water were demonstrated to possess excellent radical-scavenging activity, ferrous ion chelating ability, lipid peroxidation inhibition activity, and antioxidative enzyme activity [20,21,22,23,24,25,26,27,28,29]. 

Previous studies have reported that kefir water possesses high antioxidant compounds including phenolic acids, flavonoids, and organic acids with abilities to scavenge free radicals [30,31]. Kefir was also reported to attenuate oxidative stress-induced neuronal damages and spinal cord injury and could be a promising candidate for neuronal protection [32]. Guven et al. [32] revealed that kefir pre-treatment significantly decreased oxidative stress and neuronal degeneration (p<0.05) as a neuroprotective agent. Moreover, the kefir group had significantly higher neurological deficit scores than the ischemia group. 

Although the antioxidant, anti-apoptotic, and neuroprotective activities of kefir water cultured in different parts of the world have been reported [20,21,22,23,24,25,26,27,28,29,30,31,32,33,34,35], there is no study made on kefir water cultured in Malaysia for its neuroprotective effects in NDD. This study investigated the neuroprotective potential of six different kefir water samples cultured in Malaysia against H_2_O_2_-induced cytotoxic effects on differentiated human neuronal cells and studied their cellular and molecular morphological features to provide a better insight into how kefir provides its health benefits. To date, this will be the very first neuroprotection study reported using kefir grains from Malaysia.

## 2. Materials and Methods

### 2.1. Preparation Kefir Water Culture

Kefir grains A, B, E, and F were obtained from a local store known as Kefir and Kombucha in Kuala Lumpur, Malaysia (https://www.kefirandkombucha.com, accessed on 1 June 2021); Kefir grain C was obtained from another local store named MyKefir World in Kuala Lumpur, Malaysia (http://mykefirworld.com, accessed on 1 June 2021); and Kefir grain D was obtained from the Amrita store in Kuala Lumpur, Malaysia (http://www.amritakefir.com/ or https://www.facebook.com/AmritaKefir/, accessed on 1 June 2021). Kefir grain A originated from France, whereas Kefir grains B, E, and F originated from Tibet. The differences among the three kefir grains from Tibet were: Kefir grain B was adapted to the acidic environment with the use of 3 tablespoons of vinegar during fermentation but was fermented without vinegar for this experiment; Kefir grain E was fermented with the addition to 3 tablespoons of vinegar; and Kefir grain F was the natural Tibetan grains alone. However, the origin of Kefir grains C and D is unknown. Kefir water was prepared by adding 18 g of each kefir grain sample into six different glass vessels, each containing 50 g raw organic sugar and 500 mL mineral water. The glass vessels were labelled accordingly (Kefir A to F) and fermented at room temperature for 24 h. Upon fermentation, kefir grains were filtered out from the fermented medium using 110 mm filter paper (Whatman, Eastleigh, Hampshire, England). The kefir water was then freeze-dried using Freezone Freeze Dry Systems (Labconco, Kansas, MO, USA). The freeze-dried kefir waters (A to F) were diluted in complete media (50 mg/mL) and stored at 4 °C until used.

### 2.2. Antioxidant Analysis of Kefir Water

#### 2.2.1. Total Phenolic Content

The total phenolic contents (TPCs) of kefir water samples were determined using the Folin–Ciocalteu method. Briefly, 0.2 mL of kefir water samples (50 mg/mL) or gallic acid standard was added to 1 mL of diluted Folin–Ciocalteu’s phenol solution and incubated for 5 min. Then, 0.8 mL of 7.5% Na_2_CO_3_ solution was added and incubated at room temperature for 2 h in the dark. After incubation, the absorbance was read at 765 nm using an ELISA plate reader (Bio-Tek Instruments, Winooski, VT, USA). TPCs of the samples were expressed in µg gallic acid equivalents (µg GAE/ µL). This experiment was performed in triplicates.

#### 2.2.2. Total Flavonoid Content

The total flavonoid contents (TFCs) of kefir water samples were measured following a method described by Saeed et al. [36] with some adjustments. Nine microlitres of 5% NaNO_3_ was mixed with 150 µL of kefir water samples (50 mg/mL) or catechin standard and incubated for 6 min in the dark. Then, 9 µL of 10% AlCl_3_ was added and incubated for 5 min. After that, 60 µL of 1M NaOH and 72 µL of distilled water were added and mixed well by vortexing. The absorbance was then read at 430 nm using an ELISA plate reader (Bio-Tek Instruments, Winooski, VT, USA). The TFCs of the samples were expressed as µg catechin equivalents (µg CAT/µL). This experiment was performed in triplicates. 

#### 2.2.3. 2,2′-Diphenyl-1-picrylhydrazyl Radical Assay

The abilities of kefir water to scavenge 2,2′-diphenyl-1-picrylhydrazyl (DPPH) free radicals were determined according to the method described by Thaipong et al. [37] with some modifications. The DPPH stock solution was prepared by mixing 24 mg DPPH powder in 100 mL methanol and stored at −20 °C until used. The working DPPH solution was prepared by mixing 10 mL DPPH stock solution in 45 mL methanol. Then, 250 µL of DPPH working solution was allowed to react with 50 µL of kefir water samples (50 mg/mL) or ascorbic acid standard for 30 min on an orbital shaker in the dark. The absorbance was then measured using an ELISA plate reader (Bio-Tek Instruments, Winooski, VT, USA) at 515 nm. The DPPH scavenging activity (percentage of inhibition) of kefir water samples were calculated using the following equation:

(1) $$\mathrm{DPPH}\;\mathrm{Scavenging}\;\mathrm{Activity}\;\%=\frac{{\mathrm{Abs}}_{\mathrm{negative}\;\mathrm{control}}{-\mathrm{Abs}}_{\mathrm{extract}/\mathrm{standard}}}{{\mathrm{Abs}}_{\mathrm{negative}\;\mathrm{control}}}\times 100\%$$

#### 2.2.4. Ferric Reducing Antioxidant Power Assay

The ferric reducing antioxidant power (FRAP) assay was performed according to Thaipong et al. [37] with slight modifications. The stock solutions of 300 mM acetate buffer (0.1168 g C_2_H_9_NaO_5_ dissolved in 1 mL C_2_H_4_O_2_ and diluted in 62.5 mL distilled water), 10 mM TPTZ (4.5 mg TPTZ in 5 mL 40 mM HCL), and 20 mM FeCl_3_.6H_2_O solution was used to prepare the FRAP working solution by the ratio of 10:1:1, respectively. The FRAP working solution was warmed at 37 °C prior to usage. Then, 250 µL of FRAP working solution was mixed with 50 µL of kefir water samples or ascorbic acid standard. The sample mixtures were shaken on an orbital shaker for 30 min in the dark. Distilled water was used as a blank. The change in the sample colour were then measured at 593 nm using an ELISA plate reader (Bio-Tek Instruments, Winooski, VT, USA). This experiment was performed in triplicates.

### 2.3. Cell Lines and Cell Culture

The human neuroblastoma (SH-SY5Y) cells were purchased from ATCC (ATCC^®^ CRL-2266™) and maintained according to the protocol described by Ismail et al. [11]. The SH-SY5Y cells were grown in a complete culture medium containing a mixture (1:1) of Dulbecco’s Modified Eagle’s medium (DMEM) and Ham’s nutrient mixture F-12 (F-12), which was supplemented with 10% fetal bovine serum, 1% non-essential amino acids, and 50 μg/mL gentamicin. Cells were maintained at 37 °C under 5% CO_2_ and 95% air. Since SH-SY5Y has neuronal-like characteristics, it was fully differentiated into neuronal cells with the appropriate trans-retinoic acid (RA) concentration, and thus is a suitable model for neuroprotection research.

### 2.4. Differentiation of SH-SY5Y Cells

The SH-SY5Y cells were differentiated according to the protocol described by Lopes et al. [38]. Briefly, the cells were seeded at a density of 2 x 10^5^ cells/well in 6-well plates. After 24 h of incubation, the spent media was substituted with 2 mL DMEM and F-12 media containing 3% heat-inactivated FBS and 10 μM RA (differentiation media) in the dark and kept in a 5% CO_2_ incubator at 37 °C. The differentiation media of the cells were changed every day for seven days. After seven days, the RA-induced differentiation was examined under contrast using an inverted light fluorescence microscope (Zeiss Axio Vert A1, Jena, Germany) equipped with an image acquisition system (AxioCam MRm, Jena, Germany). Multiple independent images of the differentiated SH-SY5Y were captured. An immunocytochemistry assay was performed for further confirmation of the SH-SY5Y differentiation. Here, the expression of Tuj-1, neuron-specific class III β-tubulin was detected using NL557- conjugate antibody.

### 2.5. Immunocytochemistry (ICC) Assay 

An immunocytochemistry (ICC) assay was conducted to further confirm the differentiation of the SH-SY5Y cells into full neuronal-like cells by RA, according to the protocol described by Jaafaru et al. [12]. The SH-SY5Y cells were seeded into 24-well culture plates at a density of 2 × 10^4^ cells/mL and were allowed to attach. After 24 h, the cells were differentiated as described above. Upon differentiation, the cells were washed three times with cold phosphate buffer saline (0.01 M phosphate buffer, 0.137 M sodium chloride and 0.0027 M potassium chloride), pH 7.4, followed by 30 min incubation with fixation solution (4% Paraformaldehyde, 1 M NaOH and PBS) at 25 °C. Then, the cells were washed three times with PBS. The cells were subsequently incubated with a permeation (0.2% Triton X-100 in 1xPBS) and a blocking (5% bovine serum albumin, 0.1% Triton X-100 in PBS) solution for 15 and 30 min, respectively, at 25 °C, accompanied by washing at each stage. The Tuj-1 antibody for Class III β-tubulin (a cytoplasmic neuron-specific protein) was prepared in a ratio of 1:200 blocking solution and incubated on cells at 4 °C overnight. The following day, the cells were washed three times with cold PBS and incubated with NL557 secondary antibody conjugate in 1:200 ratios in the dark for 2 h at 25 °C. Lastly, the cells were washed with PBS three times and incubated with DAPI dye (nuclear counterstaining dye) for 5 min before viewing under an inverted light fluorescence microscope (Zeiss Axio Vert A1, Jena, Germany) equipped with an image acquisition system (AxioCam MRm, Jena, Germany) where multiple independent images were captured.

### 2.6. MTT Assay

The ability of kefir water to protect the SH-SY5Y cells from H_2_O_2_-induced oxidative damage and cytotoxicity was evaluated using a 3-(4,5-dimethylthiazol-2-yl)-2,5-diphenyltetrazolium bromide (MTT) assay. The MTT assay was performed according to the modified protocol described by Ismail et al. [11]. The SH-SY5Y cells were seeded in 96-well culture plates at a density of 2 × 10^5^ cells/mL and differentiated for seven days, as mentioned above, prior to treatment. To examine the possible toxic effects of kefir water samples, the cells were treated with kefir water A to F over a concentration range of 10 to 1 mg/mL for 24, 48, and 72 h. Then, 20 µL MTT was added to all the wells and allowed to incubate in the dark at 37 °C for 4 h. After the incubation, the reagent was replaced with 200 µL of DMSO to solubilise the formazan crystal formed in each well. The amount of MTT formazan product was determined by measuring absorbance at 540 nm using a Microplate reader (Bio-Tek Instruments, Winooski, VT, USA). Based on this cytotoxicity analysis, Kefir B and Kefir C were selected for the subsequent experiments because they demonstrated the highest and lowest cell viability, respectively. The same method was used to evaluate the cytotoxic effect of H_2_O_2_. The cells were treated with 1000 µM of H_2_O_2_ then serial diluted to 31.25 µM to examine the IC50 of H_2_O_2_. For the determination of neuroprotective effects, the differentiated cells were pre-treated with the serially diluted respective kefir samples (1 to 10 mg/mL) for 24, 48, and 72 h and challenged by 190 μM (IC50) H_2_O_2_ for 24 h. The concentration for the untreated SH-SY5Y cells were stated as 0 mg/mL. The MTT followed by the DMSO solutions were added, and the absorbance was read as described above. The experiments were performed in triplicates.

### 2.7. Acridine Orange and Propidium Iodide (AOPI) Double Staining

The AOPI assay was performed according to Ismail et al. [11] with some modifications. The SH-SY5Y cells were seeded at a density of 2 × 10^5^ cells/mL in 6-well plates and differentiated as described above. The cells were pre-treated with Kefir B and Kefir C separately for 48 h and then exposed to 190 µM H_2_O_2_ for 24 h. The cells were trypsinised, washed twice with PBS and re-suspended in 100 µL of PBS. A combination of 5 µL acridine orange (1 mg/mL) and 5 µL of propidium iodide (1 mg/mL) was mixed with 10 µL of cell suspension and incubated for 10 min in the dark at room temperature. Then, 10 µL of the stained cell mixture was transferred to a glass slide and examined under an inverted fluorescence microscope (Zeiss Axio Vert A1, Jena, Germany) equipped with an image acquisition system (AxioCam MRm, Jena, Germany). Multiple independent images were captured.

### 2.8. Annexin V-FITC Apoptosis Analysis 

Cell apoptosis was detected using a PI/Annexin V-FITC apoptosis detection kit (BD Biosciences, San Jose, CA, USA) based on the protocol enclosed in the kit. The SH-SY5Y cells were seeded at a density of 2 × 10^5^ cells/mL in 6-well plates (as described by Ismail et al. [11]), differentiated, and pre-treated with Kefir B and C samples separately for 48 h, followed by 190 µM H_2_O_2_ exposure for 24 h as described previously. The cells were trypsinised, washed with PBS twice, and resuspended with 1X binding buffer (0.1 M HEPES/NaOH pH 7.4, 1.4 M NaCl and 25 mM CaCl_2_). The cells were stained with 5 µL Annexin V-FITC and 5 µL PI and incubated at room temperature for 15 min in the dark. After incubation, 400 µL of 1X binding buffer was added to the cell mixture prior to the analysis by NovoCyte flow cytometer (ACEA Biosciences Inc., San Diego, CA, USA). The experiment was conducted in triplicates.

### 2.9. Scanning Electron Microscopy (SEM)

The ultrastructural morphological features of the surface of SH-SY5Y were examined using scanning electron microscopy. The protocol for the SH-SY5Y cell preparation prior to SEM was followed according to the method described by Jaafaru et al. [12]. In brief, the SH-SY5Y cells were seeded at a density of 1 x 10^6^ cells in T25 mL flasks and differentiated as described above. The cells were pre-treated with Kefir B and C for 48 h and then challenged with 190 µM H_2_O_2_ for 24 h. At the end of the treatment, the cells were trypsinised and washed with PBS twice. The protocol for SEM was obtained at the Microscopic Unit, Institute of Bioscience, Universiti Putra Malaysia. The PBS-washed cells were fixed with 4% glutaraldehyde for 6 h and 1% osmium tetraoxide for 2 h. Between these treatments, the cells were washed three times with a 0.1 M sodium cacodylate buffer at 10-min intervals. After discarding the fixatives, the cells were dehydrated with a series of acetone concentrations (35, 50, 75, and 95%). Further dehydration was carried out three times using 100% acetone. The cells were dried off for 30 min on a critical dryer. After mounting, the dried cell pellets were immediately coated with gold particles and viewed under SEM (JSM 6400, Peabody, MA, USA). Multiple independent images were captured at different magnifications.

### 2.10. Transmission Electron Microscopy (TEM)

The protocol for TEM was obtained at the Microscopic Unit, Institute of Bioscience, Universiti Putra Malaysia and the SH-SY5Y cells were prepared according to Jaafaru et al. [12]. The SH-SY5Y cells were seeded at a density of 1 × 10^6^ cells in T25 mL flasks and differentiated. The cells were pre-treated with Kefir B and C for 48 h and then challenged with 190 µM H_2_O_2_ for 24 h. At the end of the treatment, the cells were trypsinised and washed with PBS twice. The cells were fixed with 4% glutaraldehyde and 1% osmium tetraoxide followed by dehydration using a series of acetone concentrations as described above. A mixture of acetone and resin (1:1 ratio) was used to infiltrate the cells for 1 h, 1:3 for 2 h, and 100% resin overnight. The infiltrated cell was put into a resin-filled beam capsule for embedment. The samples were polymerised for two days at 60 °C in the oven. The specimen was cut into 1 μM thick sections by an ultra-microtome. The sections were stained with toluidine, and the thickness of the specimen was reduced to 60 ± 90 nm. Then, the thinner sections were stained using uranyl acetate for 15 min and lead for 10 min before viewing under TEM (JEM-2100F, Peabody, MA, USA). Multiple independent images were captured at different magnifications.

### 2.11. Quantitative Real-Time PCR (qPCR) Analysis

#### 2.11.1. RNA Extraction

Total RNA was isolated from the SH-SY5Y cells pre-treated with Kefir B and C and induced with H_2_O_2_ using the Qiagen RNEasy Mini Kit (Qiagen, Hilden, NRW, Germany) according to the manufacturer’s protocol. The concentration and purity of the RNA were quantified using a nanospectrophotometer (Thermo Fisher Scientific Inc., San Diego, CA, USA). RNA purity was assured to be within the ratios of 1.8–2.0 at A_260_/A_280_.

#### 2.11.2. Primer Design

Primers were designed using the Integrated DNA Technologies (IDT) system (https://sg.idtdna.com/PrimerQuest/Home/Details/0_2, accessed on 1 June 2020) using Homo sapien sequence from the National Centre for Biotechnology Information GenBank Database (NCBI Genbank). The selected genes of interest and house-keeping genes are listed in Table 1. Both the forward and reverse primers carry the universal tag sequences in addition to the complementary nucleotides of the targeted genes. The primers were obtained from First Base Ltd. (Seri Kembangan, Selangor, Malaysia). The forward and reverse primers were diluted in 1X TE buffer to 200 nM and 500 nM concentrations, respectively.

#### 2.11.3. qPCR Analysis

Maxima First Strand complementary DNA (cDNA) synthesis kit (Thermo Fisher Scientific Inc., San Diego, CA, USA) was used to convert the total RNA (1 µg) into cDNA, which were then loaded into a thermal cycler (Labnet, USA). The cDNA was then used to quantify the selected gene expressions (Table 1) on the Eco^TM^ Real-Time PCR System (Illumina Inc., San Diego, CA, USA) using a Maxima SYBR Green qPCR Master Mix (Thermo Fisher Scientific Inc., San Diego, CA, USA). The mixture was prepared by mixing 12.5 µL of Maxima SYBR Green qPCR Master Mix, 0.3 µM of forward and reverse primers and ≤ 500 ng of cDNA. Nuclease-free water was used to top up the mixture up to 25 µL. The mixture was then run using the Eco^TM^ Real-Time PCR System (Illumina Inc., San Diego, CA, USA) platform and the reaction was set at 95 °C for 10 min, 40 cycles of denaturation at 95 °C for 15s, and annealing/extension at 55–60 °C for 15 to 30s.

### 2.12. Statistical Analysis

All data are presented as mean ± SD. The data were evaluated by one-way ANOVA using SPSS software (SPSS Inc., Chicago, IL). Differences between the means were assessed using Tukey’s HSD post-hoc test. Statistical significance was considered at the probability (p) value less than 0.05.

## 3. Results

### 3.1. In Vitro Antioxidant Analysis

The antioxidant activity of the kefir water samples were determined through the TPC, TFC, DPPH, and FRAP assays. As shown in Table 2, Kefir B has the higher TPC (1.96 ± 0.54 µg GAE/µL) and TFC (1.09 ± 0.02 µg CAT eq/µL) levels compared to other kefir samples. The TPC level in Kefir B is significantly high when compared to Kefir C (0.064, 95% CI (0.007 to 0.121), *p* = 0.025). In addition, the DPPH assay shows that only a 0.45 ± 0.06 mg/mL concentration is required for Kefir B to scavenge 50% of 2,2-diphenyl-1-picrylhydrazyl free radical activity, unlike other kefir samples that require higher concentrations. The FRAP assay also resulted in higher antioxidant activity in Kefir B sample with 19.68 ± 0.11 FRAP mM FRAP eq/ 50 µL.

### 3.2. Differentiation of SH-SY5Y Cells into Neuronal Cells

**Figure 1:**
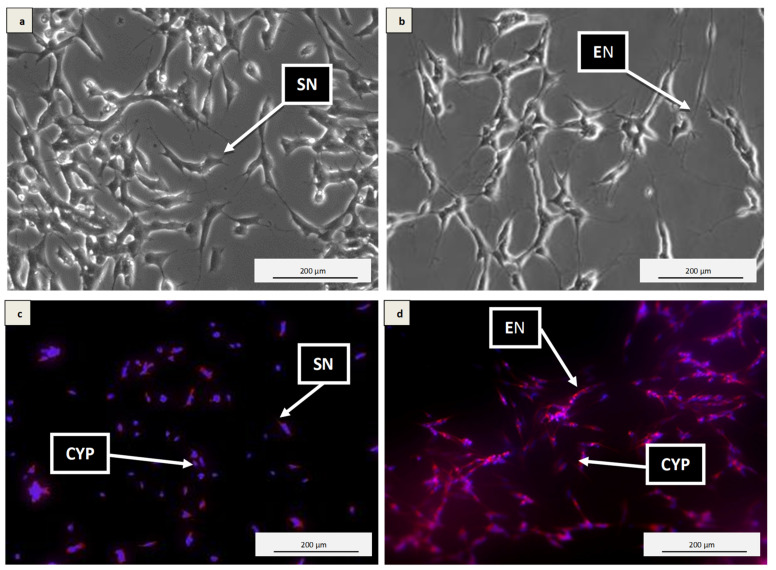
Micrographs of SH-SY5Y cell differentiation by 10 μM RA. (**a**) Undifferentiated SH-SY5Y cells cultured in 10% heat-inactivated FBS complete growth media for 7 days and viewed under phase contrast, (**b**) Differentiated SH-SY5Y cells cultured in 3% heat-inactivated FBS complete growth media containing 10 μM RA for 7 days and viewed under phase contrast, and (**c** and **d**) expressed tuj-1 in both neurites and cytoplasm. Extended neurites = EN, short neurites = SN, and cytoplasm = CYP. Magnification (40x).

The differentiation process of the SH-SY5Y cells was confirmed by immunocytochemistry. Based on the observation under the phase contrast microscopy, short neurites in the undifferentiated SH-SY5Y cells (Figure 1a) and extended neurites upon the SH-SY5Y cells’ differentiation (Figure 1b) were clearly visible. In the undifferentiated SH-SY5Y cells, the intensity of the red fluorescence was weak and was found only in a few cells (Figure 1c). However, in the differentiated SH-SY5Y cells (Figure 1d), the red fluorescence intensity was markedly increased in both the neurites and cytoplasm. Higher red fluorescence intensity indicates increased tuj-1 expressions in the differentiated SH-SY5Y cells, confirming the differentiation process.

### 3.3. Effect of Different Kefir Water Samples on SH-SY5Y Cells Viability

**Figure 2:**
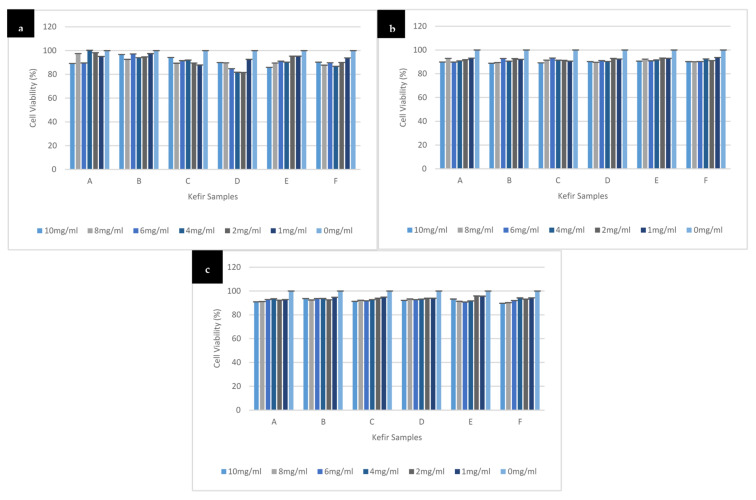
Cytotoxicity of kefir water samples (A–F) on differentiated SH-SY5Y cells at different concentrations (1 to 10) mg/mL. Displayed kefir water treatment at (**a**) 24 h, (**b**) 48 h, and (**c**) 72 h. The experiment was carried out in triplicates and values are presented in means ± SD.

As shown in Figure 2, the kefir water (A–F) treated cells were viable across all concentrations used (1 to 10) mg/mL and at all-time points (24 h, 48 h, and 72 h). The result showed that the percentage of cell viability of the kefir waters (A–F) were above 80% at all time points. Thus, the highest concentration (10 mg/mL) of kefir water was chosen for the subsequent experiments.

A one-way ANOVA was conducted to determine any significant changes in the cell viability upon treatment with kefir water (A–F) across all concentrations (1 to 10) mg/mL and at all-time points (24 h, 48 h, and 72 h). The data showed a significant decrease in the cell viability for Kefir A, F(6,14) = 9.134, *p* < 0.0005; Kefir D, F(6,14) = 3.941, *p* = 0.016; Kefir E, F(6,14) = 7.600, *p* = 0.001; and Kefir F, F(6,14) = 6.255, *p* =0.002. There is no statistically significant difference in the cell viability for Kefir B, F(6,14) = 1.565, *p* = 0.229 and Kefir C, F(6,14) = 2.197, *p* = 0.106 treatments compared to the control. The data for 48 h of kefir treatments showed significant reduction in the cell viability for all the concentrations treated when compared to the control: Kefir A, F(6,14) = 10.670, *p* < 0.0005; Kefir B, F(6,14) = 10.437, *p* < 0.0005; Kefir C, F(6,14) = 12.366, *p* < 0.0005; Kefir D, F(6,14) = 36.850, *p* < 0.0005; Kefir E, F(6,14) = 23.162, *p* < 0.0005; and Kefir F, F(6,14) = 19.772, *p* < 0.0005; as well as for 72 h of kefir treatments: Kefir A, F(6,14) = 7.724, *p* = 0.001; Kefir B, F(6,14) = 9.057, *p* < 0.0005; Kefir C, F(6,14) = 31.120, *p* < 0.0005; Kefir D, F(6,14) = 12.702, *p* < 0.0005; Kefir E, F(6,14) = 17.417, *p* < 0.0005; and Kefir F, F(6,14) = 23.079, *p* < 0.0005. Tukey HSD post-hoc analysis revealed that the decrease in cell viability in Kefir B (3.261, 95% CI (−6.380 to 12.903)), Kefir C (5.813, 95% CI (−7.388 to 19.014)), and Kefir D (10.067, 95% CI (−5.904 to 26.039)) treatments at 10 mg/mL for 24 h is not significant (*p* = 0.900, *p* = 0.739, and *p* = 0.376, respectively) when compared to the control. Kefir treatments for 48 h and 72 h at 10 mg/mL when compared to the control showed significant decrease in cell viability: Kefir A 48 h (10.175, 95% CI (4.969 to 15.382), *p* < 0.0005), 72 h (9.040, 95% CI (3.736 to 14.344), *p* = 0.001); Kefir B 48 h (11.182, 95% CI (5.613 to 16.750), *p* < 0.0005), 72 h (6.338, 95% CI (2.185 to 10.491), *p* = 0.002); Kefir C 48 h (10.875, 95% CI (5.999 to 15.751), *p* < 0.0005), 72 h (8.618, 95% CI (6.037 to 11.200), *p* < 0.0005); Kefir D 48 h (9.754, 95% CI (6.910 to 12.597), *p* < 0.0005), 72 h (7.915, 95% CI (4.312 to 11.519), *p* < 0.0005); Kefir E 48 h (9.994, 95% CI (6.507 to 13.480), *p* < 0.0005), 72 h (6.796, 95% CI (2.981 to 10.612), *p* < 0.0005); and Kefir F 48 h (9.818, 95% CI (5.944 to 13.691), *p* < 0.0005), 72 h (10.209, 95% CI (6.771 to 13.647), *p* < 0.0005).

### 3.4. Effect of Different Kefir Water Samples on H_2_O_2_-Induced Cell Death in SH-SY5Y Cells

**Figure 3:**
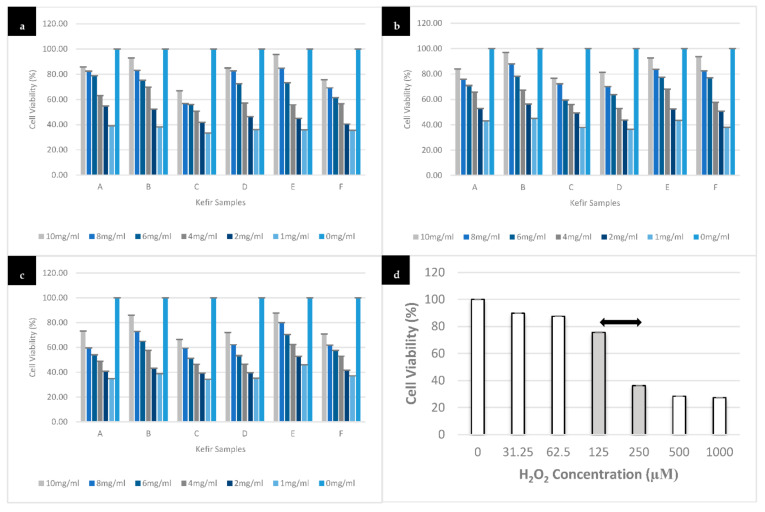
Concentration-dependent viability of the differentiated SH-SY5Y cells pre-treated with kefir water samples (1 to 10 mg/mL). (**a**) Display 24 h, (**b**) 48 h, and (**c**) 72 h plus 24 h of 190 µM H_2_O_2_ exposure, whereas (**d**) is an H_2_O_2_-induced (31.25 to 1000) µM cytotoxic analysis used in this study with IC50 = 190 µM at 24 h. The experiment was carried out in triplicates and values are presented in means ± SD.

Hydrogen peroxide induced cell death of 50% of the cell population at 190 µM in 24 h (Figure 3d). This H_2_O_2_ concentration was therefore selected to challenge the kefir water pre-treated differentiated cells in the subsequent experiments. Likewise, the kefir pre-treated and H_2_O_2_-induced differentiated cells were also analysed accordingly. The kefir water (A–F) pre-treatment protected the cells against H_2_O_2_-induced cytotoxic effects across the experimental period (Figure 3a–c). Kefir water pre-treatment at 10 mg/mL for 48 h signifies the highest cell viability, Kefir B (2.963, 95% CI (−11.275 to 17.201), *p* = 0.990) and Kefir F (6.407, 95% CI (−3.501 to 16.316), *p* = 0.350), followed by Kefir E (7.298, 95% CI (−11.703 to 26.300), *p* = 0.836), Kefir A (16.064, 95% CI (−8.928 to 41.056), *p* = 0.356), Kefir D (18.676, 95% CI (10.081 to 27.271), *p* < 0.0005), and lastly Kefir C (23.245, 95% CI (7.563 to 38.928), *p* = 0.003). Hence, kefir treatment at 10 mg/mL for 48 h was chosen as the working concentration and treatment duration throughout the experiments. Moreover, kefir pre-treatment that resulted in the highest (Kefir B) and lowest (Kefir C) cell viability was chosen in the subsequent experiments to compare their effects as a neuroprotective agent.

A one-way ANOVA was conducted to determine any significant changes in the cell viability upon pre-treatment with kefir water (A–F) across all concentrations (1 to 10) mg/mL and at all-time points (24 h, 48 h, and 72 h) plus 190 µM H_2_O_2_ for 24 h. The data for 24 h of kefir treatment showed that the decrease in cell viability in Kefir A (F(6,14) = 52.829), Kefir B (F(6,14) = 58.647), Kefir C (F(6,14) = 249.606), Kefir D (F(6,14) = 189.363), Kefir E (F(6,14) = 59.328), and Kefir F (F(6,14) = 32.790) were significant (*p* < 0.0005) when compared to the control. Similarly, data for 48 h of kefir treatment showed that the decrease in cell viability in Kefir A (F(6,14) = 13.511), Kefir B (F(6,14) = 49.916), Kefir C (F(6,14) = 39.658), Kefir D (F(6,14) = 154.611), Kefir E (F(6,14) = 27.599), and Kefir F (F(6,14) = 126.643); and the decrease in cell viability in 72 h of kefir treatment in Kefir A (F(6,14) = 212.158), Kefir B (F(6,14) = 268.637), Kefir C (F(6,14) = 228.136), Kefir D (F(6,14) = 248.773), Kefir E (F(6,14) = 78.069), and Kefir F (F(6,14) = 558.361) were significant (*p* < 0.0005) when compared to the control. Tukey HSD post-hoc analysis revealed that the decrease in cell viability in Kefir B (7.193, 95% CI (−6.582 to 20.968)), Kefir E (9.685, 95% CI (−5.262, 24.632)), and Kefir F (18.994, 95% CI (-0.082 to 38.070)) treatments at 10 mg/mL for 24 h is not significant (*p* = 0.578, *p* = 0.348, and *p* = 0.051, respectively) when compared to the control. The reduction of cell viability in Kefir A (16.064, 95% CI (−8.928 to 41.056)), Kefir B (2.963, 95% CI (−11.275 to 17.201)), Kefir E (7.298, 95% CI (−11.703 to 26.300)), and Kefir F (6.407, 95% CI (−3.501 to 16.316)) for 48 h at 10 mg/mL is not significant (*p* = 0.356, *p* = 0.990, *p* = 0.836 and *p* = 0.350, respectively) when compared to the control. The decrease in cell viability upon kefir treatments (A–F) for 72 h at 10 mg/mL is significant when compared to the control: Kefir A (26.933, 95% CI (19.632 to 34.234), *p* < 0.0005), Kefir B (14.116, 95% CI (7.621 to 20.611), p < 0.0005), Kefir C (33.604, 95% CI (26.565 to 40.642), *p* < 0.0005), Kefir D (27.925, 95% CI (21.099 to 34.750), *p* < 0.0005), Kefir E (12.223, 95% CI (1.696 to 22.750), *p* = 0.019), and Kefir F (29.277, 95% CI (25.003 to 33.551), *p* < 0.0005).

### 3.5. Acridine Orange and Propidium Iodide (AO/PI) Double Staining Assay

**Figure 4:**
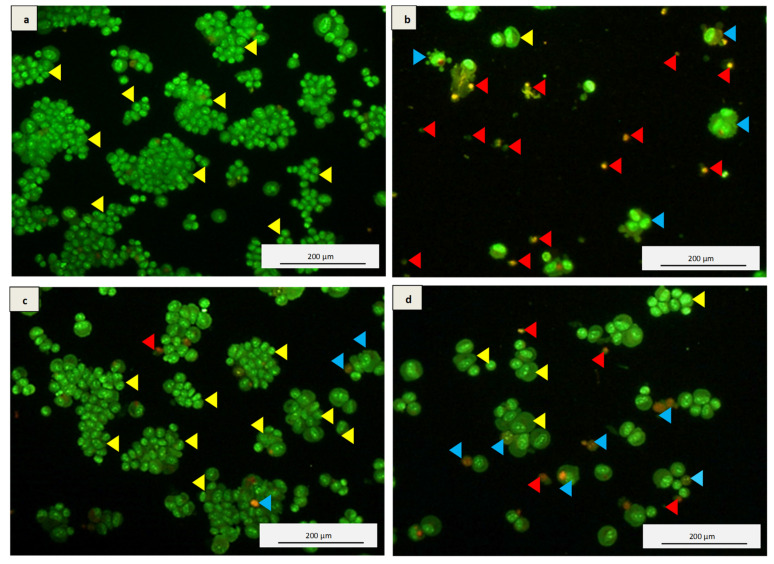
Micrographs of acridine orange (AO, green) and propidium iodide (PI, red) double staining on differentiated SH-SY5Y cells. (**a**) Untreated differentiated SH-SY5Y cells, (**b**) SH-SY5Y cells challenged with H_2_O_2_ for 24 h, (**c**) 48 h Kefir B pre-treated plus 24 h H_2_O_2_-exposed SH-SY5Y cells, (**d**) 48 h Kefir C pre-treated plus 24 h H_2_O_2_-exposed SH-SY5Y cells (yellow arrowhead: viable; blue arrowhead: apoptosis; red arrowhead: necrosis). The images were taken independently in multiple times, and 40x magnification was used.

Acridine orange (AO) and propidium iodide (PI) double staining were performed to observe the differences between the untreated and kefir water (B and C) pre-treated differentiated cells using fluorescence microscopy. Viable cells were signified by the green stained nucleus (Figure 4a–d) of the differentiated cells, whereas the cells that are unprotected from H_2_O_2_-induced oxidative damage by means of undergoing apoptosis were stained in red to orange in the same figures. The results showed that the pre-treatment with Kefir B and Kefir C waters demonstrated increased cell viability with a high percentage of green-stained nuclei. However, the percentage of cells stained in red-orange in Kefir C pre-treatment was higher compared to Kefir B.

### 3.6. Annexin V-FITC Assay

**Figure 5:**
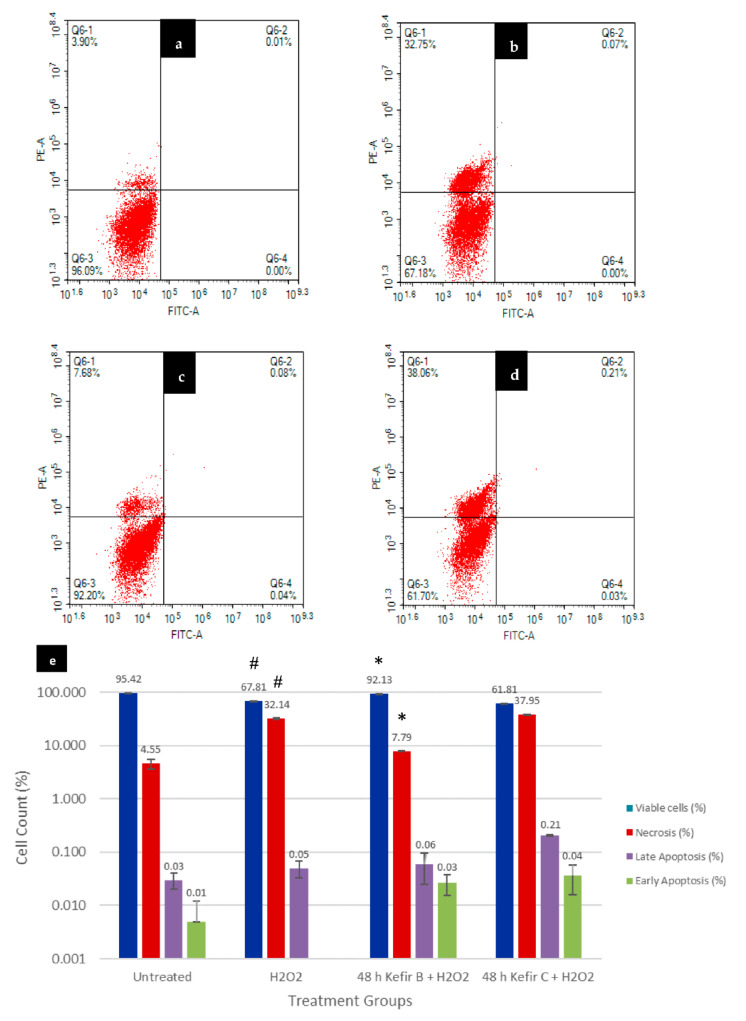
Flow cytometry Annexin V-FITC analysis of differentiated SH-SY5Y cells where (**a**) untreated SH-SY5Y cells, (**b**) 24 h H_2_O_2_-exposed cells, (**c**) 48 h Kefir B pre-treated plus 24 h H_2_O_2_-exposed cells, (**d**) 48 h Kefir C pre-treated plus 24 h H_2_O_2_-exposed cells, and (**e**) quantification analysis of cells at death. The experiment was carried out in triplicates and values are presented in means ± SD. # *p* < 0.0005 versus untreated, * *p* < 0.0005 versus H_2_O_2_. A one-way ANOVA was conducted to determine the significance of 48 h Kefir B and Kefir C pre-treatment on H_2_O_2_-induced cellular apoptosis. The one-way ANOVA test showed statistically significantly difference in the percentage of viable cells F(3,7) = 2438.178, *p* < 0.0005, and necrosis cells F(3,7) = 2534.183, *p* < 0.0005 found in the dot plot. Tukey HSD post-hoc analysis revealed that the increase in the percentage of viable cells (%) when compared to H_2_O_2_ treated cells to untreated control cells (27.607, 95% CI (25.911 to 29.302)), H_2_O_2_ treated cells to Kefir B pre-treated plus H_2_O_2_-induced cells (24.313, 95% CI (22.797 to 25.830)), and Kefir C pre-treated plus H_2_O_2_-induced cells to Kefir B pre-treated plus H_2_O_2_-induced cells (30.320, 95% CI (28.803 to 31.837)) were statistically significant (*p* < 0.0005), as well as the increase in the percentage of necrotic cells (%) when compared to untreated control cells to H_2_O_2_ treated cells (27.592, 95% CI (25.934 to 29.249), *p* < 0.0005), Kefir B pre-treated plus H_2_O_2_-induced cells to H_2_O_2_ treated cells (24.350, 95% CI (22.868 to 25.832), *p* < 0.0005), and H_2_O_2_ treated cells to Kefir C pre-treated plus H_2_O_2_-induced cells (5.813, 95% CI (4.331 to 7.296), *p* < 0.0005).

Annexin V-FITC and PI stains were used to evaluate cellular apoptosis upon Kefir B and Kefir C pre-treatment plus H_2_O_2_ exposure. Cells in the lower-left quadrant of the dot plot (Annexin-V-/PI-) and the lower-right quadrant (Annexin-V+/PI-) were indicated as healthy, viable cells and cells undergoing early apoptosis, respectively. On the other hand, cells in the upper-right quadrant (annexin-V+/PI+) were undergoing late apoptosis, whereas cells in the upper-left quadrant (annexin-V-/PI+) were undergoing necrosis (Figure 5a–d). The results showed that 32.14% of H_2_O_2_-challenged cells underwent necrosis and 0.05% underwent late apoptosis compared to the untreated control cells (Figure 5e). In the Kefir C pre-treated plus H_2_O_2_-induced cells, 37.95%, 0.21%, and 0.04% of cells underwent necrosis, late, and early apoptosis, respectively (Figure 5e). Interestingly, in comparison to the H_2_O_2_-challenged cells, the Kefir B pre-treated plus H_2_O_2_-induced cells resulted in a significantly lower percentage of necrotic cells (7.79%, p < 0.0005) and a remarkable increase in cell viability (92.13%, p < 0.0005) similar to the untreated control cells was observed in the same figure (Figure 5e).

### 3.7. Scanning Electron Microscopy (SEM)

**Figure 6:**
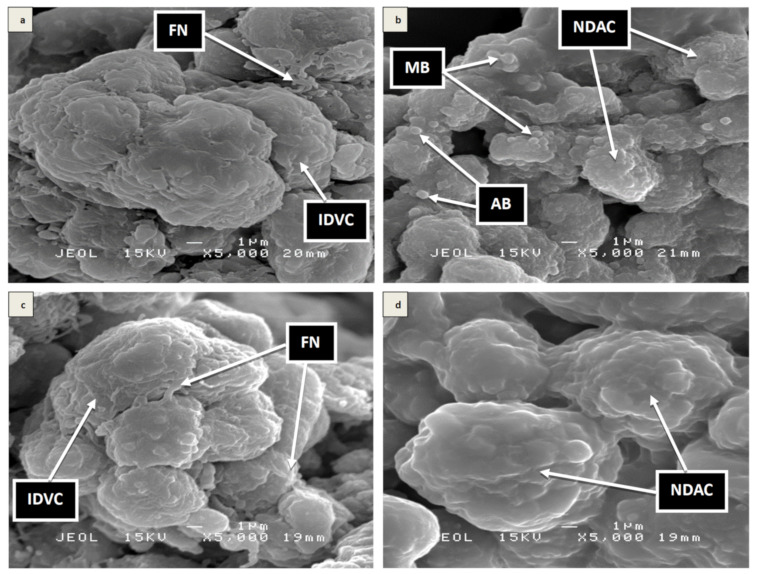
Surface morphological analysis of the differentiated SH-SY5Y cells by scanning electron microscopy. (**a**) Untreated differentiated SH-SY5Y cells, (**b)** 24 h H_2_O_2_-challenged cells, (**c**) 48 h Kefir B pre-treated plus 24 h H_2_O_2_-exposed cells, and (**d**) 48 h Kefir C pre-treated plus 24 h H_2_O_2_-exposed cells. FN = folded neurites, IDVC = intact differentiated viable cells, NDAC = neurite disrupted apoptotic cells, AB = apoptotic body, MB = membrane blebbing. Magnification (x 5000).

SEM revealed an interesting outcome on the cellular surface ultrastructural analysis of the differentiated SH-SY5Y cells pre-treated with and without Kefir B or Kefir C plus H_2_O_2_. In the H_2_O_2_-exposed cells (Figure 6b), neurite disruption, apoptotic bodies, and membrane blebbing were observed. Likewise, in the Kefir C pre-treated plus H_2_O_2_-exposed cells (Figure 6d), neurite-disrupted apoptotic cells were found. However, the surface features of the differentiated SH-SY5Y cells pre-treated with Kefir B plus H_2_O_2_ (Figure 6c) appeared intact with viable integrated cytosol and folded neurites, which was similar to the untreated normal control cells (Figure 6a).

### 3.8. Transmission Electron Microscopy (TEM)

**Figure 7:**
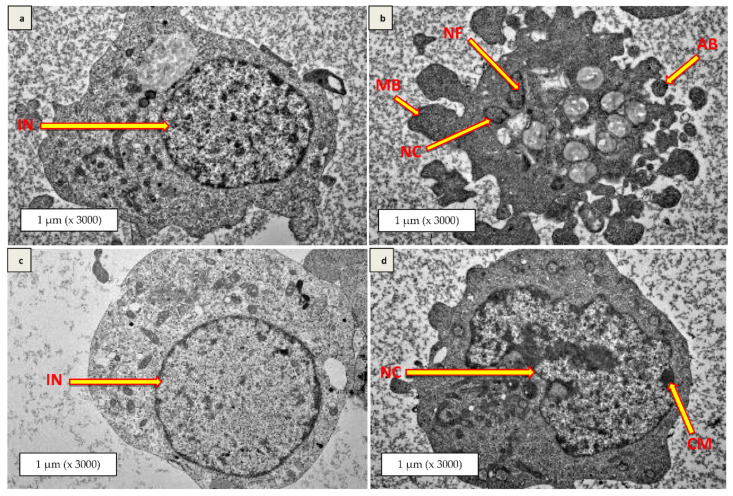
Internal morphological analysis of differentiated SH-SY5Y cells by transmission electron microscopy. (**a**) Untreated differentiated SH-SY5Y cells, (**b**) 24 h H_2_O_2_-exposed cells, (**c**) 48 h Kefir B pre-treated plus 24 h H_2_O_2_-exposed cells, and (**d**) 48 h Kefir C pre-treated plus 24 h H_2_O_2_-exposed cells. IN = intact nucleus, NC = nuclei convolution, CM = chromatin margination, MB = membrane blebbing, AB = apoptotic bodies, NC = nuclear condensation, NF = nuclear fragmentation. Magnification (x 3000).

The internal morphological features of the differentiated SH-SY5Y cells, assessed using TEM, revealed interesting outcomes. It was found that the H_2_O_2_-treated cells showed morphological aberration where nuclear condensation, nuclear fragmentation, apoptotic bodies, and membrane blebbing were obvious (Figure 7b). Likewise, the Kefir C pre-treated plus H_2_O_2_-induced cells (Figure 7d) resulted in nuclear convolution, chromatin margination, and condensation. However, these features were absent in the Kefir B pre-treated plus H_2_O_2_-exposed cells (Figure 7c) and the untreated control SH-SY5Y cells (Figure 7a).

### 3.9. qPCR Analysis

**Figure 8:**
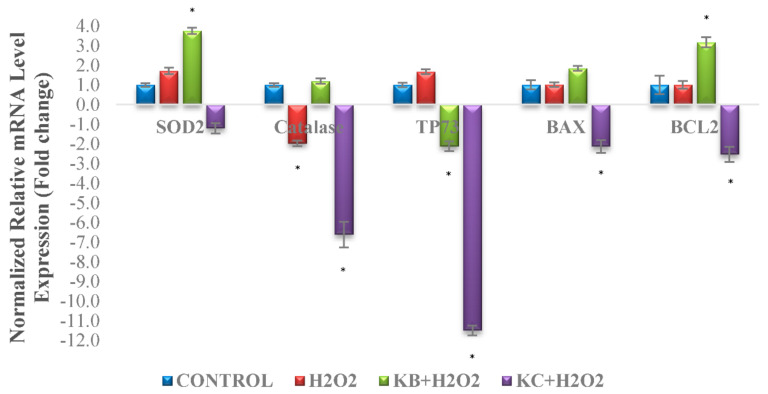
Expression levels of the superoxide dismutase (SOD) 2, catalase, Tp73, Bax, and Bcl-2 genes in SH-SY5Y cells. mRNA levels of SOD2, catalase, Tp73, Bax, and Bcl-2 genes following treatment with 10 mg/mL Kefir B and Kefir C for 48 h and subsequent treatment with 190 µM H_2_O_2_ for 24 h, in comparison to untreated and 190 µM H_2_O_2_ treated controls.

The mRNA levels of two antioxidants genes (SOD2 and catalase) and three apoptotic related genes (Tp73, Bax, and Bcl-2) were studied using an Eco Real-Time PCR analysis system. The complete list of the target and housekeeping genes is shown in Table 1. As shown in Figure 8, 190 µM of the H_2_O_2_ treatment significantly downregulated the mRNA levels of catalase and upregulated the levels of SOD2 and Tp73, whereas, for Bax and Bcl-2 genes, the expression levels were found to be similar compared to the control. The Kefir B plus H_2_O_2_ treatment significantly downregulated the expression of the Tp73 gene while upregulating SOD2 and Bcl-2 in comparison to the H_2_O_2_-treated cells. In the case of the Kefir C plus H_2_O_2_ treatment, the expressions of catalase, Tp73, Bax, and Bcl-2 were significantly downregulated.

## 4. Discussion

The findings from this study revealed an insight into the neuroprotection ability of kefir water samples against H_2_O_2_-induced oxidative stress in differentiated human neuroblastoma (SH-SY5Y) cells. Researchers have identified various bacterial strains from probiotic-rich foods that possessed natural antioxidant activities, and one of the best sources of probiotics found in the kefir culture is the Lactobacillus species. Studies have reported the ability of the Lactobacillus species in producing natural antioxidant compounds that prevent oxidative stress damages due to free radical production in the host [32,33,39,40,41,42,43]. Table 2 shows the analysis of antioxidant potencies (TPC, TFC, FRAP, and DPPH) of the six water kefir samples made from kefir grains available in Malaysia. Phenolic acids and flavonoids are the general classes of polyphenols. The presence of phenolic and flavonoid compounds in food is beneficial for human consumption as they provide high antioxidant and anti-mutagenicity effects. Based on Table 2, kefir B has a higher TPC (1.96 ± 0.54 µg GAE/µL) and TFC (1.09 ± 0.02 µg CAT eq/µL) contents compared to the other kefir samples. Moreover, the FRAP value of kefir water B was 19.68 mM FRAP equivalent/ 50 µL, and 0.45 mg/mL of kefir water B was required to reduce 50% (IC50) of radical scavenging (DPPH) activity. Although, there were no significant differences in the antioxidant activities among the six kefir samples, Kefir B showed greater antioxidant activities compared to the other kefir samples, especially Kefir C, which had the lowest antioxidant potentials. Commonly, water kefir was found to possess lower scavenging activities than kefir fermented in milk. Since the starting material used for kefir fermentation was only sugar water, no protein interaction was involved in producing more antioxidants, and milk alone contains high antioxidant activities [21]. A study by Liu et al. [21] demonstrated that there was no significant difference in the chelating effects by ferrous ions among the fermented and unfermented milk. The study showed an immediate increase in scavenging activities of kefir grains inoculated in milk or soy milk, suggesting that the antioxidant components from kefir grains were transferred and interacted with the peptides deriving from milk and soy milk.

SH-SY5Y cells are the most popular model used in current neuroscience research, particularly for neurodegenerative diseases, including Alzheimer’s disease. SH-SY5Y cells are dopaminergic neuronal cells. Treatment with differentiation inducers such as retinoic acid (RA) helps them develop neuronal properties such as neural extension and the expression of particular neuron-specific markers [44,45,46,47]. However, differentiated SH-SY5Y cells have lower susceptibility to cytotoxic effects of various compounds, thereby enhancing their stability compared to undifferentiated SH-SY5Y [48,49,50,51]. In differentiated SH-SY5Y cells, H_2_O_2_-induced cytotoxicity overwhelms endogenous defensive mechanism system by triggering a cascade of reactions that results in oxidative stress conditions and cell death [11,12,52]. On the other hand, exogenous antioxidants deplete reactive species generations and prevent oxidative damage in the cells, thus increasing cell survival [11,12,53]. The MTT result showed how different kefir samples (A–F) and various concentrations enhanced differentiated neuron’s viability in a time-dependent manner. However, the maximum potential was exhibited by Kefir B (10 mg/mL) at 48 h as showed in Figure 3b, signifying the high ability to reduce the susceptibility of the cells against the exogenous cytotoxic agent. Studies have reported that oxidative damage triggers a cascade of reactions that causes adverse effects on antioxidant mechanistic pathways [52,53]. Such effects can be prevented by exogenous antioxidants, but, when introduced in larger quantities, they tend to increase the cells’ sensitivity to stimuli with subsequent cell death [53]. Based on both the antioxidant and cytotoxicity analyses, Kefir B and Kefir C were chosen for the subsequent experiments as they demonstrated the highest and lowest antioxidant potentials and cell viability, respectively.

The neuroprotective effects of Kefir B and Kefir C on cell viability were assessed by the AO/PI double staining method. AO is permeable to the cellular membrane; thus, it penetrates the viable cells and stains the cellular nucleus green. On the other hand, PI is a membrane-impermeable intercalating agent, which can only be taken up by cells with a disrupted cell membrane and stains the cellular nucleus red [4,12]. The Kefir B pre-treatment prior to H_2_O_2_-induced cytotoxicity indicated the highest level of protection with a greater number of the green-stained nucleus compared to H_2_O_2_ treatment alone, which affects the cells (stained orange or red) as observed in Figure 4. Hydrogen peroxide induced cytotoxicity resulted in destructive cellular morphological changes such as cell shrinkage, apoptotic bodies, membrane blebbing, and cell debris. Thus, Kefir B demonstrated enhanced neuroprotective activity against H_2_O_2_-induced cellular death compared to Kefir C.

Moreover, the Kefir B pre-treatment protected the differentiated neuronal cells from undergoing early and late apoptosis or necrosis induced by H_2_O_2_ exposure, as shown in Figure 5. The result suggests that Kefir B could keep the lipid asymmetry membrane intact and prevent the translocation of phosphatidylserine (PS) to cytoplasm. This PS translocation is due to the internal reactive species generation in cells that are exposed to oxidative stress conditions, which promotes the disruption of asymmetrical membrane status [54,55]. The result obtained from PI/Annexin V-FITC analyses signified that necrosis is the predominant event that occurred when the differentiated SH-SY5Y cells were treated with 190 µM H_2_O_2_ for 24 h. Likewise, Takeda et al. [55] reported that H_2_O_2_ treatment most frequently resulted in necrosis in every experimental condition tested. Hence, Kefir B is the most potent agent that prevents necrosis against H_2_O_2_-induced neuronal cell death.

An ultrastructural surface analysis on the differentiated SH-SY5Y cells by SEM revealed the ability of the Kefir B pre-treatment prior to H_2_O_2_-induced cytotoxicity to protect cell surface structures and preserve membrane integrity including the extended neurites of differentiated neurons (Figure 6). The folded neurites in the Kefir B pre-treated plus H_2_O_2_-induced cells were very similar to the untreated neuron cells. However, the neurites in the H_2_O_2_ treated cells were disrupted, and apoptotic bodies and membrane blebbing were also observed [13]. The Kefir C pre-treated plus H_2_O_2_-induced cells showed no effect on the differentiated neuronal cells. Similar findings were reported by Jaafaru et al. [12] where H_2_O_2_ treatment disrupted the neurites while glucomoringin-isothiocyanate treatment protects membrane integrity and the extended neurites of the neuronal cells. This indicates that the Kefir B pre-treatment provides high neuroprotection against H_2_O_2_-induced oxidative stress.

Furthermore, the internal morphological analysis of H_2_O_2_-induced differentiated neuronal cells using TEM (Figure 7) resulted in nuclear condensation and fragmentation, and apoptotic bodies and membrane blebbing are features in cells undergoing apoptosis [56,57]. The Kefir B pre-treatment prevents the occurrences of such events and protects the neuronal cells with an intact nucleus and membrane integrity. On the contrary, the Kefir C pre-treated plus H_2_O_2_-induced cells showed features typical to that of apoptosis. Hence, Kefir B possessed a robust neuroprotection ability by inhibiting the internal reactive species generation mechanisms.

The observation of apoptosis for the H_2_O_2_-induced cells contradicts the earlier claims of PI/Annexin V-FITC analyses where necrosis was observed as the predominant event that occurred upon H_2_O_2_ induction. However, other studies have reported that apoptosis and necrosis can occur simultaneously [56,57,58,59]. This may be due to the interconnection of the downstream death signaling pathways [59]. Wang et al. [60] reported that necrotic cell death induced by oxidative stress involves the activation of the apoptosis-associated caspase-8/Bid pathway. Another study by Leist et al. [61] demonstrated that cells undergo necrotic cell death when apoptosis is blocked. However, the final form of cell death is dependent on the initial inducers, cell type, and cell microenvironment.

Apoptosis is majorly regulated by a series of caspase cascades. It consists of two main pathways: intrinsic pathways, which are mitochondrial-dependent, and extrinsic pathways, which are often activated by death receptors [62]. In the intrinsic pathway, the membrane of the mitochondria depolarises, permeabilises, and begins to rupture, releasing pro-apoptotic factors that result in the formation of apoptosomes, which subsequently activates the caspase cascade [62,63]. On the other hand, apoptosis via the extrinsic pathway is activated by the death receptors on the surface of the cell membranes, which then finally activates caspase cascade [63]. Bax is a pro-apoptotic member of the Bcl-2 protein family that functions to regulate the mitochondrial membrane potential [61,62]. This result in the secretion of cytochrome C and subsequent activation of the caspase cascade causing apoptosis [63,64]. In addition, the tumour protein p73 (Tp73) is a member of the p53 family and its overexpression can trigger p53-related genes and induce apoptosis [65,66]. SOD2 and catalase are the antioxidant genes crucial for the antioxidant defence systems in H_2_O_2_-induced differentiated neuronal cells.

The results showed a significant decrease in the expression of catalase in the H_2_O_2_-induced cells. In the differentiated SH-SY5Y cells, H_2_O_2_ upregulates the endogenous antioxidant levels to protect cells from oxidative damage by activating the intracellular defence mechanisms. However, in excess, the defence mechanisms will fail to overcome the H_2_O_2_ effects and will lead to apoptosis and/or necrosis [67]. The Kefir B treatment resulted in a significant increase in the SOD2 expression compared to the H_2_O_2_-induced neuronal cells. This suggests that Kefir B is capable of protecting the neuronal cells by activating the intracellular antioxidant defence mechanisms to counter oxidative stress by H_2_O_2_. Likewise, a study by Ismail et al. [11] showed an increase in antioxidant gene expressions upon treatment with an oryzanol-rich fraction prior to H_2_O_2_ induction, suggesting an increase in the antioxidant defence system in SH-SY5Y. On the other hand, the Tp73 apoptosis regulating gene expression was significantly reduced by the Kefir B treatment, whereas it was enhanced in the H_2_O_2_-induced cells. This further shows the potency of Kefir B in protecting the neuronal cells from undergoing cell death due to oxidative damage. However, the expressions of Bax and Bcl-2 in the H_2_O_2_-induced cells were similar to those of the untreated cells. This might be due to the cells undergoing apoptosis and the activation of necrosis pathways simultaneously [56,57,58,59]. When apoptotic cells undergo secondary necrosis, it changes the plasma membrane permeability by forming membrane pores and then ruptures, releasing the intracellular contents [68]. Nevertheless, the Bcl-2 anti-apoptotic gene expression was enhanced in the Kefir B pre-treated plus H_2_O_2_-induced cells. This finding is in agreement with Raguenez et al. [69], where Bcl-2 upregulation in FGF1-differentiated SH-SY5Y cells is associated with neuronal survival.

Kefir B revealed a robust neuroprotection ability in differentiated SH-SY5Y cells through the abolishment of internal reactive species generation mechanisms. This might be due to the bioactive compounds found in Kefir B which resulted in greater potential in reducing activity in FRAP and scavenging activity in DPPH, with enhanced flavonoid and phenolic contents determined during fermentation compared to Kefir C. This was clear through cell viability assay, AO/PI double staining assay, PI/Annexin-V FITC analysis, surface and internal morphological analyses using SEM and TEM, respectively, and antioxidant (SOD2 and catalase) and apoptotic (Tp73, Bax and Bcl-2) gene expressions studies where Kefir B showed the most potency in eradicating intercellular ROS. The study showed that the different antioxidant capacities found in Kefir B and C produced different effects on the SH-SY5Y cells. This might be due to the different microbial composition found in kefir grain B and C. Depending on the dominant microbial strains found in both the kefir grains, certain strain-specific bioactive compounds will be produced. These bioactive components will provide different potential effects or health benefits according to the nature and function of the bioactive components. This study shows that Kefir B has potential antioxidant effects, while the antioxidant activity of Kefir C is weaker. Nevertheless, Kefir C might hold a different bioactive property that has to be further investigated through the identification of the microbial composition in the kefir grains and the detection of available bioactive compounds responsible for such action.

## 5. Conclusions

The findings from the present study highlighted the neuroprotective activity of kefir water in H_2_O_2_-induced, differentiated SH-SY5Y cells. This could be facilitated by the antioxidant and anti-apoptotic activities of the kefir water evaluated from antioxidant assays, cell viability, fluorescence microscopy, and flow cytometry analyses. Interestingly, the results have revealed the strength of Kefir water B in protecting the differentiated neuronal cells membrane and the internal structural integrity from the exposure to oxidative damage by H_2_O_2_, signifying its ability in conserving neurons from degeneration due to oxidative stress. Hence, future research will identify the underlying mechanisms on how kefir water provides such neuroprotection, the essential bioactive compounds and microorganisms responsible for such actions, and their potential effects among the kefir grains studied. Moreover, future study is also required to understand the link between apoptosis and the necrosis pathway and its action during intracellular reactive species generation in neuronal cells.

## Figures and Tables

**Table 1:** Details of the gene, accession number, and the reverse and forward primer sequences used in Eco Real-Time PCR analysis system.

Gene	Accession Number	Forward (5′-3′)	Reverse (5′-3′)
**SOD2**	NM_000636	AAACTGAGAGCAAAGAATGGAG	CCACAAGCACAGAAATAAAGG
**Catalase**	NM_001752	GGTAACCCAGTAGGAGACAAAC	CGAGATCCCAGTTACCATCTTTC
**Tp73**	NM_005427.4	AGCAGCCCATCAAGGAGGAGTT	TCCTGAGGCAGTTTTGGACACA
**Bax**	NM_001291428	CAAGAAGCTGAGCGAGTGT	CAGTTGAAGTTGCCGTCAGA
**Bcl-2**	NM_000633	TTGACAGAGGATCATGCTGTACTT	ATCTTTATTTCATGAGGCACGTT
**ACTB**	NM_001101	AGAGCTACGAGCTGCCTGAC	AGCACTGTGTTGGCGTACAG
**GAPDH**	NM_002046	GGATTTGGTCGTATTGGGC	TGGAAGATGGTGATGGGATT

**Table 2:** Antioxidant assays and the total phenolic and flavonoid content for kefir samples.

Kefir Samples	TPC (µg GAE/µL Kefir Sample) #	TFC (µg CAT eq/µL Kefir Sample) #	DPPH Free Radical Scavenging Assay (mg/mL) *	FRAP (mM FRAP eq/50 µL Kefir Sample) **
Kefir A	1.11 ± 0.64	1.01 ± 0.01	0.76 ± 0.32	18.32 ± 0.10
Kefir B	1.96 ± 0.54 ***	1.09 ± 0.02	0.45 ± 0.06	19.68 ± 0.11
Kefir C	0.68 ± 0.28	0.92 ± 0.01	0.80 ± 0.01	15.25 ± 0.49
Kefir D	1.43 ± 0.37	0.96 ± 0.01	0.58 ± 0.03	17.87 ± 0.38
Kefir E	1.14 ± 0.12	0.98 ± 0.01	0.49 ± 0.16	18.11 ± 0.23
Kefir F	ND	0.91 ± 0.01	0.68 ± 0.16	17.24 ± 0.25

Note: TPC, total phenolic content; TFC, total flavonoid content; DPPH, di[phenyl]-[2,4,6-trinitrophenyl] iminoazanium; FRAP, ferric reducing antioxidant power assay; ND, not detected. # Value expressed as mean ± standard deviation. *Expressed as IC50 (mg/mL), calculated from Trolox standard curve (y = 0.7167x − 3.2129, r^2^ = 0.9972), with smaller values signifying higher antioxidant capacity. ** Expressed as mM FRAP eq/µL sample, calculated from ferrous sulphate standard curve (y = 0.733x + 0.1247, r^2^ = 0.9975). *** *p* < 0.05. A one-way ANOVA was conducted to determine the significance in the antioxidant activity in Kefir A-F. The data showed no significant difference in the level of antioxidant in TFC (F(5,12) = 89.591, *p* > 0.05), DPPH (F(5,12) = 2.380, *p* = 0.101), and FRAP (F(5,12) = 1.048, *p* = 0.434) assays. The data showed a significant difference in the total phenolic content (F(5,12) = 20.399, *p* < 0.0005). Further evaluation using Tukey HSD post-hoc analysis revealed that the total phenolic content in Kefir B is significantly increased (0.064, 95% CI (0.007 to 0.121), *p* = 0.025) when compared to Kefir C.

## Data Availability

The data presented in this study are available on request from the corresponding author.
